# Notes and News

**Published:** 1891-10-31

**Authors:** 


### Notes and News.

COLLECTED AND COMMUNICATED.

*Truth* last week attacked St. Bartholomew's Hospital because one of the blocks holding eight wards is closed during the alteration of the sanitary plans. Only four months ago *Truth* was declaring that the whole of St. Bartholomew's Hospital ought to be closed. The damsel which adorns the title page of the paper in question ought obviously to be holding one of those reversible mirrors one side of which contradicts the expression reflected by the other side.

An instance of the utility of the Post Office for begging purposes is given. A lady advertised in a newspaper asking persons to send 3d. each in aid of the Bishop of Bedford's Whitechapel Refuge Fund, and to induce two friends to do the same. She subsequently removed from the address without giving notice of the change, and the replies accumulated at the Returned Letter Office to such an extent, that, when her new residence was discovered, no fewer than 16,268 letters containing £191 were awaiting delivery.

Thanks to the Irish Dispensaries, Committee meeting reports are not all dull reading—Cork no longer entertains us, but Castle Pollard (West Meath) steps into the breach. For reasons that do not appear, a systematic attack has been made upon the district doctor, Dr. King. He was charged with having assaulted a messenger who came to summons his aid on his wife's behalf, also with having left the district when urgently needed. A Local Board Enquiry was instituted. During the proceedings the alleged assaulted was enquired for. He was reported not present. Mr. Harrington, for the defence, immediately instructed the police to search for him in the public-houses. As the evidence proceeded the wisdom of this direction was apparent. Boylan, for whom search was made, was found to have been convicted of 22 offences since 1885. Further enquiry brought out the fact that the assault was unwitnessed and had been affirmed on Boylan's statement alone. He was seen in a state of intoxication the same night, and all witnesses admitted that Boylan with a black eye would have been no strange sight. Owing to this unsatisfactory evidence the charge fell through, or was the other one substantiated. All the proceedings are not reported as five gentlemen present were seized with a desire to speak at the same time. The Church Militant, assisted throughout the meeting, as is usual in all Irish proceedings.

The Rev. J. T. Christie, British Chaplain of Genoa and Savona, writes, calling attention to the good work done by the Church Mission to Seamen. He says: I have two seamen's institutes under my charge; one in Genoa, and one in Savona. They both have a resident reader, who visits the ships for about eight hours daily, distributing tracts, leaflets, and useful literature, and inviting the men to come to the institutes, where everything is provided for their comfort and social recreation, and where on certain fixed days during each week concerts, temperance, and prayer meetings are held, and special services conducted by the Chaplain. The men are followed up by correspondence and commended to other missions when possible; 14,864 seamen have visited our institute in Genoa last year, and 9,476 our institute at Savona; and the £500 a year (roughly speaking) required to support these institutes is made up entirely by the voluntary contributions of the friends of the mission and the seamen themselves. This year we require a considerable increase of funds.

The pulse of generous charity beats high in Birmingham. We are informed that the Misses Stokes, of Edgbaston, have, as a memorial of their late brother, Mr. Alfred Stokes, offered to provide the whole of the purchase money—£7,500—for the acquisition of the building near Llandudno which it has been resolved to acquire for the purpose of the Hospital Saturday Convalescent Home. Mr. Alfred Stokes was well known as one of the largest coal merchants in Birmingham, and was in business on the Old Wharf for more than half a century, and was deservedly respected by those who knew him. We have no doubt the Committee of the Hospital Saturday Fund will be greatly encouraged by so munificent a mark of sympathy with their movement, which will relieve their fund from the burden entailed by borrowing the purchase money, and will enable the acquisition of the property to be immediately completed. The works necessary for its adaptation to its intended use can then be promptly carried into effect, and the Home opened before the date of the next Hospital Saturday collection.

It would be hard to find a brighter or more healthy locality on which to build a hospital than the site that has been chosen by Mrs. Harvey for her children's convalescent home at Shanklin. The home is divided into three blocks, which are connected with each other by wide passages. One block is for the girls, the next for the boys, and the third is devoted to the offices, &c. There is a chapel 70 feet long, with a corridor opening into it. Two long slit windows open into it from the ward set apart for eight of the worst cases, so that the sick children may hear the service. The dining-room for the children is 60 feet long, and broad in proportion, and here all of them (boys and girls) dine; the dinner tables only take up one side of it, and the rest of it will be used as a play-room for the boys. In the girls' block there will also be a ward and a sitting-room adjoining it, with sliding doors, for eight children of the better class, who will pay at the rate of one guinea a-week. There are also six rooms for ladies who may like to come here for a time to take part in the work of the house or for the good of their own health. They also are to pay one guinea a week. The accommodation for the working staff of twenty Sisters is very complete and comfortable. The building is to be lighted by electricity. In fact, all modern ideas have been made use of which can add to comfort or save trouble. There are "shoots" from the upper regions for soiled linen, others for dust and housemaids' sweepings, while lifts also economise labour. The home is built to accommodate 100 persons (staff and patients). The children will be admitted by letters from the subscribers of one guinea, who can send a child there for one month in the year.

